# Night-time versus daytime surgical outcomes in chronic subdural hematomas: a post hoc analysis of the FINISH randomized trial

**DOI:** 10.1007/s00701-024-06302-9

**Published:** 2024-10-22

**Authors:** Elias Oulasvirta, Oula Knuutinen, Pihla Tommiska, Riku Kivisaari, Rahul Raj, the FINISH study group, Abdirisak Ahmed, Tarmo Areda, Jiri Bartek, Tomasz Czuba, Nils Danner, Antti-Pekka Elomaa, Janek Frantzén, Ilkka Haapala, Joonas Haapasalo, Juuso Heikkilä, Minttu Hellman, Henna Henttonen, Nora Huuska, Teppo LN Järvinen, Henna-Kaisa Jyrkkänen, Aku Kaipainen, Olli-Pekka Kämäräinen, Hanna Kämppi, Milla Kelahaara, Riku Kivisaari, Nikolai Klimko, Oula A Knuutinen, Timo Koivisto, Tommi Korhonen, Janne Koskimäki, Anselmi Kovalainen, Xenia Kuparinen, Dan Laukka, Martin Lehecka, Kai Lehtimäki, Ville Leinonen, Kimmo Lönnrot, Antti Luikku, Teemu Luostarinen, Teemu Luoto, Janne Luotonen, Lauriina Lustig-Tammi, Henna-Riikka Maanpää, Jenni Määttä, Timo Möttönen, Eliisa Netti, Laura Nevaharju-Sarantis, Mika Niemelä, Tero Niskakangas, Mette Nissinen, Ville Nurminen, Minna Oinas, Teemu Ollonen, Anna Östberg, Elias Oulasvirta, Krista Pantzar, Katri Piilonen, Anni Pohjola, Markus Polvivaara, Jussi P Posti, Rahul Raj, Linnea Rajala, Jonas Ranstam, Minna Rauhala, Behnam Rezai Jahromi, Miika Roiha, Ilkka Saarenpää, Antti Sajanti, Henrikki Salmi, Jarno Satopää, Christoph Schwartz, Niina Shemeikka, Pia Sorto, Simo Taimela, Sami Tetri, Tuomo Thesleff, Pihla Tommiska, Maarit Tuomisto, Nuutti Vartiainen, Ville Vasankari, Jyri Virta, Mikko Visuri, Paula Walle, Frederick A Zeiler

**Affiliations:** 1 Department of Neurosurgery, Helsinki University Hospital and University of Helsinki, Bridge Hospital, HUS, Haartmaninkatu 4, Po. Box 340, 00029 Helsinki, Finland; 2 Department of Neurosurgery, Oulu University Hospital, Oulu, Finland

**Keywords:** Intracranial hemorrhages, Time-to-treatment, Surgical timing, Chronic subdural hematoma, Nigh-time surgery, Daytime surgery

## Abstract

**Objective:**

The optimal timing of surgical intervention for chronic subdural hematomas (CSDH), specifically night-time versus daytime, remains a subject of debate, with concerns about the potential impact of circadian timing on surgical outcomes. This study evaluated the association between the timing of burr-hole drainage for CSDH and postoperative outcomes, comparing night-time and daytime surgeries.

**Methods:**

In a post-hoc analysis of the FINISH trial, we included adult patients with symptomatic unilateral or bilateral CSDH who underwent burr-hole drainage between January 2020 and August 2022. Night-time surgery was defined as procedures starting between 23:00 and 06:00, with daytime surgeries occurring between 06:01 and 22:59. The primary outcome was functional outcome at six months post-surgery, assessed using the modified Rankin Scale (mRS), with favorable outcomes defined as an mRS of 0–3. Secondary outcomes included mortality, reoperation rates, and adverse events within six months.

**Results:**

Our analysis of 589 patients (83% daytime surgery, 17% night-time surgery) revealed no significant differences in baseline characteristics. The unadjusted analysis suggested a higher rate of favorable functional outcomes in the night-time surgery group than in the daytime group (94% vs. 86%, *p* = 0.037). Mortality, adverse events, and reoperation rates were similar in the groups. Adjusted logistic regression analyses, accounting for potential confounders, indicated that night-time surgery was not associated with a higher risk of unfavorable functional outcomes compared to daytime surgery.

**Conclusions:**

Our findings suggest that night-time surgery versus daytime surgery is not associated with worse postoperative outcomes. These findings challenges the traditional preference for daytime CSDH surgery and emphasizes the potential for flexibility in surgical scheduling to optimize patient care in CSDH management.

**Supplementary Information:**

The online version contains supplementary material available at 10.1007/s00701-024-06302-9.

## Introduction

The correlation between circadian timing and surgical outcomes is a debated issue, with many studies suggesting the possibility of increased complications and adverse events during night-time surgeries [2, 4, 9, 10, 12, 15, 23]. It is well established that both alertness and cognitive functions are compromised during night hours or in the event of sleep-deprivation [5, 17, 21]. Moreover, hospital resources, including equipment, staffing, and diagnostic services are less readily available during the night, and the assistance of senior colleagues is not so accessible. While it is imperative to perform some emergency surgeries immediately to save lives and improve functional outcomes, irrespective of the time of day, not all surgical interventions are equally urgent. In such cases, it is debated whether it would be more prudent to postpone surgery until daytime when conditions would presumably be more favorable. However, it should be noted that surgical delays, even for less acute surgeries, have been associated with potentially deteriorating outcomes [1, 24].

Chronic subdural hematoma (CSDH), while not often immediately life threatening, necessitates timely management to avoid potential long-term morbidity [22]. In this context, patients with CSDH can be compared to those with hip fractures, in whome early surgical intervention is believed to improve recovery by facilitating mobilization, subsequently reducing the duration of hospital stay [6, 13, 19, 22]. It can also be argued that the cognitive demand for burr-hole drainage surgery is quite low, making its performance at night safer than other more complex surgeries.

We aimed to investigate the association between night-time versus daytime surgery (burr-hole drainage) and outcome in patients operated on for CSDH. We hypothesized that the CSDH surgical procedure would be sufficiently straightforward and simple for the time of the day not to have an effect on patient outcomes.

## Methods

### Patient population

The current study was a post-hoc analysis of the FINISH trial results [18]. The trial comprised adult patients (age ≥ 18 years) with symptomatic unilateral or bilateral CSDH undergoing burr-hole drainage between January 2020 and August 2022. Exclusion criteria were patients with prior intracranial surgery (including cerebrospinal fluid shunt), insufficient co-operation for subdural drain placement, CNS tumor interfering with operation or assessment of symptoms or outcome, acute infection requiring antibiotics, recent treatment for hematological malignancy, comatose condition, and high risk for life-threatening thrombosis when discontinuation of antithrombotic medication was not advisable (e.g. mechanical mitral or tricuspid valve, recent coronary/intracranial stent). All patients in the intention-to-treat analysis were included.

### Ethical considerations

This study was conducted and reported in accordance with the Strengthening the Reporting of Observational Studies in Epidemiology (STROBE) guidelines. We received written informed consent from the patients or their next-of-kin. The FINISH trial was approved by the ethics committee of Helsinki University Hospital (HUS/3035/2019 §238).

### Surgical procedure

Surgeries were performed under local anesthesia with few exceptions. A routine preoperative antibiotic was given according to local protocols (normally a second-generation cephalosporin 30–60 min) prior to incision. All patients underwent a burr-hole drainage procedure including a single burr-hole for access, and subdural drain placement. Half of the patients underwent subdural irrigation before the subdural drain placement and half of the patients did not undergo the irrigation. Patients from both study arms were included, as there was no difference in outcomes between the subdural irrigation and the no irrigation groups [18]. During the passive subdural drainage period (48 h ± 12), the patients were allowed to move freely and did not routinely receive prophylactic antibiotics.

### Definition of outcomes and timing of surgery

The primary outcome was functional outcome at six months from the index surgery, according to the modified Rankin Scale (mRS). Favorable functional outcome was defined as an mRS of 0–3 and unfavorable functional outcome as an mRS of 4–6. Trained research nurses and researchers assessed the mRS according to a standardised algorithm. Secondary outcomes were mortality, reoperation rate, and adverse events within six months from the index surgery. Adverse events were categorized as severe adverse event (SAE), minor adverse event (MAE), and procedure-related adverse events (PRAE).

Night-time surgery was defined as surgery starting between 23:00 and 06:00 (daytime surgery starting between 06:01 and 22:59).

### Statistical analysis

Normally distributed data are presented as means with standard deviations (SD) and non-parametric data as medians with interquartile ranges (IQR). Normally distributed data were compared between groups using a t-test and non-parametric data were compared using a Wilcoxon rank sum test. Categorical data were compared using a two-sided χ2 square test.

To assess the independent association between night-time and daytime surgery and their outcomes, we used logistic regression analysis adjusting for clinically relevant admission characteristics and baseline variables that significantly (p < 0.05) differed between night-time and daytime patients. The study center was additionally adjusted for due to observed differences in the rates of night-time and daytime surgeries. Separate logistic regression models were created to test the association between night-time and daytime surgery and their functional outcomes (mRS 0–3 vs. 4–6), mortality at six months, reoperation rate within six months, and adverse events (all adverse events [AE], SAE, MAE, PRAE). Multicollinearity was tested using a Variance Inflation Factor (VIF) test. A sensitivity analysis using ordinal logistic regression, with mRS as an ordinal outcome variable, was performed.

In all analyses, a two-tailed p-value < 0.05 was considered statistically significant. The statistical analysis was performed using Stata version 15 (StataCorp).

## Results

**Fig. 1:**
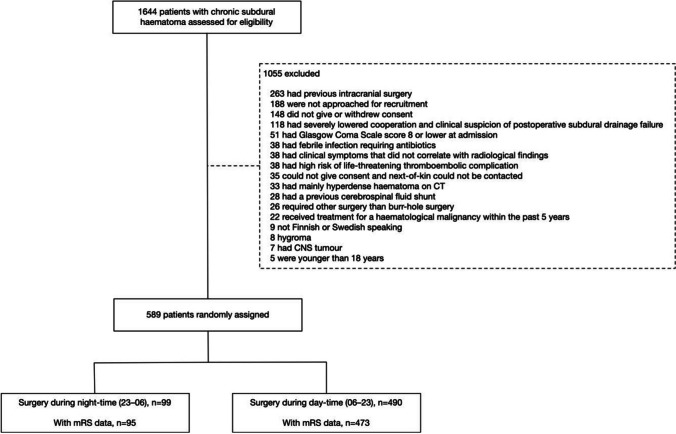
Flowchart of patient selection in the FINISH trial and subsequent post-hoc analysis of surgery timing

Our post hoc analysis of the FINISH trial comprised a total of 589 patients, with 490 (83%) undergoing daytime surgery and 99 (17%) night-time surgery for CSDH (Fig. 1). The night-time and daytime patients were well balanced in terms of baseline characteristics (Table 1). Differences in baseline characteristics between the night-time and daytime patients included cardiac arrhytmia (16% vs 27%, *p* = 0.027), preoperative speech disturbance (21% vs 30%, *p* = 0.044), preoperative use of antithrombotic medication (35% vs. 50%, *p* = 0.009), midline shift on preoperative imaging (median 7 mm vs. 6 mm, *p* = 0.009), and hematoma width (26 mm vs. 23 mm, *p* = 0.001).

**Table 1:** Baseline characteristics patients having surgery during night-time versus daytime

Characteristic	Daytime surgery *(n* = 490)	Nigh-time surgery (*n* = 99)	*P*-value
Age, yr– mean (SD)	78 (10)	77 (10)	0.856
Female sex – no./total no. (%)	138 (28%)	27 (27%)	0.857
Medical comorbidities – no./total no. (%)*			
Diabetes mellitus	112 (23%)	16 (16%)	0.141
Cardiac arrhythmia	131 (27%)	16 (16%)	0.027
Previous cerebrovascular event	74 (15%)	10 (10%)	0.194
Hypertension	289 (59%)	51 (52%)	0.170
Ischemic heart disease or peripheral artery disease	86 (18%)	14 (14%)	0.410
Cardiac valve prosthesis	2 (0.5%)	0 (0%)	0.524
Pulmonary embolism or deep vein thrombosis^†^	13 (3%)	0 (0%)	0.101
Dementia	61 (12%)	10 (10%)	0.513
History of head trauma – no./total no. (%)			0.785
Yes	368 (75%)	74 (75%)	
No	48 (10%)	8 (8%)	
Unknown	74 (15%)	17 (17%)	
Preoperative use of antithrombotic medication – no./total no. (%)			0.009
No	246 (50%)	64 (65%)	
Yes	244 (50%)	35 (35%)	
Symptoms at admission– no./total no. (%)^‡^			
Gait disturbance or falls	317 (65%)	72 (73%)	0.124
Hemiparesis	257 (52%)	60 (61%)	0.138
Speech disturbance	103 (21%)	30 (30%)	0.044
Cognitive impairment	191 (39%)	44 (44%)	0.311
Headache	125 (26%)	19 (19%)	0.182
Seizure	10 (2%)	0 (0%)	0.152
Other	41 (8%)	6 (6%)	0.440
GCS at admission – no./total no. (%)			0.368
15	392 (80%)	73 (74%)	
13–14	70 (14%)	18 (18%)	
9–12	28 (6%)	8 (8%)	
Modified Rankin Scale at admission – no./total no. (%)			0.225
1–3	328 (67%)	60 (61%)	
4–5	162 (33%)	39 (39%)	
Midline shift, mm – median (IQR)	6 (2, 9)	7 (3, 11)	0.009
Hematoma laterality§ – no./total no. (%)			0.321
Unilateral	369 (75%)	70 (71%)	
Bilateral	120 (25%)	29 (29%)	
Hematoma width, mm – mean (SD) ^¶^	23 (18, 29)	26 (22, 32)	0.001
Randomised group – no./total no. (%)			0.727
Irrigation	243 (50%)	51 (52%)	
No irrigation	247 (50%)	48 (48%)	

*GCS*, Glasgow Coma Scale; *SD*, Standard Deviation; *YR*, years

^*^one patient can have several comorbidities

^†^within 12-months before admission

^‡^one patient can have several symptoms

^¶^patient can have a hematoma not operated on (one preoperative computed tomography scan in the no irrigation group was not retrievable

sum of left and right hematoma widths

### Unadjusted outcomes

**Fig. 2:**
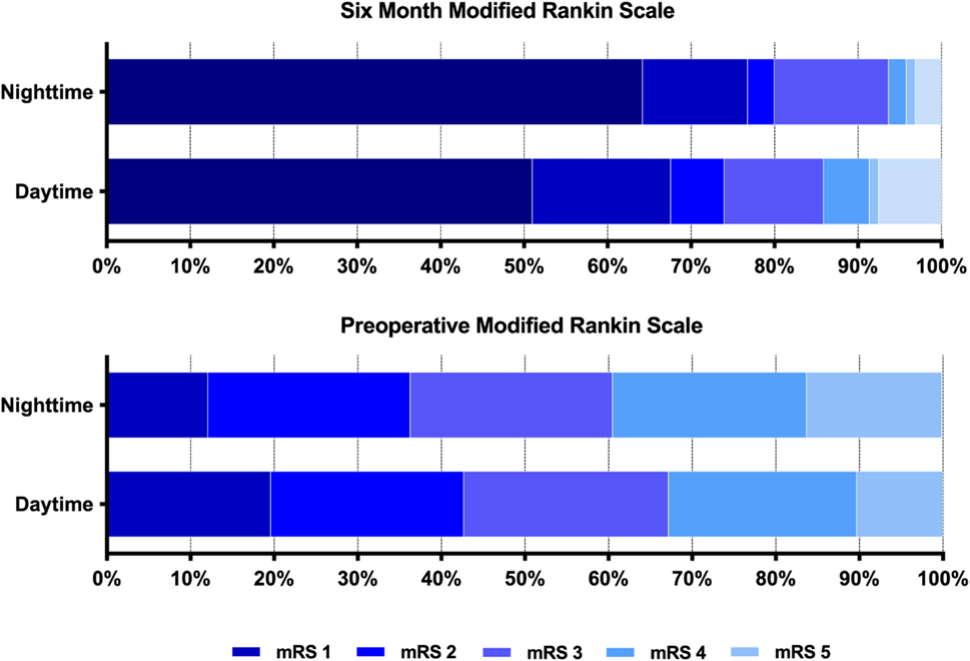
Modified rankin scale preoperatively and at six months

In the night-time surgery group, 94% of patients (*n* = 89/95) achieved a favorable functional outcome, compared to 86% (*n* = 406/473) in the daytime surgery group (*p* = 0.037). Figure 2. Six-month mortality rates did not differ significantly between the groups (3% vs. 7%, *p* = 0.115). Similarly, no statistical differences were observed in AEs (25% vs. 31%, *p* = 0.233), SAEs (12% vs. 19%, *p* = 0.094), and MAEs (14% vs. 17%, *p* = 0.489). However, PRAEs were more frequent in the daytime group (8% vs. 2%, *p *= 0.038). The reoperation rates were comparable (15% vs. 16%, *p* = 0.928) between the two groups. Table 2. A detailed description of all AEs is given in Supplementary Table 1.

**Table 2:** Unadjusted primary and secondary outcomes

Variable	Daytime surgery (*n* = 490)	Nigh-time surgery (*n* = 99)	*P*-value
Modified Rankin Scale at 6 months*, no./total no. (%)			0.037
0–3	406 (86)	89 (94)	
4–6	67 (14)	6 (6)	
Death within 6 months, no./total no. (%)	36 (7)	3 (3)	0.115
Any adverse event, no./total no. (%)	153 (31)	25 (25)	0.233
Severe adverse event	94 (19)	12 (12)	0.094
Minor adverse event	83 (17)	14 (14)	0.489
Procedure-related adverse event	38 (8)	2 (2)	0.038
Reoperation, no./total no. (%)	76 (16)	15 (15)	0.928

*Modified Rankin Scale available for 568 patients

### Adjusted outcomes

The final logistic regression analyses included the following statistically significant differences from the univariate analyses: cardiac arrhytmia comorbidity, preoperative use of antihrombotic medication, midline shift, hematoma width, and the following variables deemed clinically relevant: patient age and sex, surgery delay, preoperative Glasgow Coma Scale (GCS) score, preoperative mRS. No significant multicollinearity was encountered in any of the models (VIF ≤ 1.5).

Night-time surgery was not associated with a higher risk of unfavorable functional outcomes compared to daytime surgery (OR 0.37, 95% CI 0.13–1.06, *p* = 0.064, Table 3). In the night-time group, the risks for SAEs (OR 0.46, 95% CI 0.23–0.94, *p* = 0.032) and PRAEs (OR 0.21, 95% CI 0.05–0.90, *p* = 0.036) was significantly lower than in the daytime group.

**Table 3:** Logistic regression analysis showing association between night-time surgery versus daytime surgery and outcomes

Variable	OR (95% CI)	*P*-value
Modified Rankin Scale 6 at months 4–6 vs. 0–3	0.37 (0.13–1.06)	0.064
Death within 6 months	0.33 (0.08–1.30)	0.112
Any adverse event	0.58 (0.33–1.01)	0.054
Severe adverse event	0.46 (0.23–0.94)	0.032
Minor adverse event	0.62 (0.32–1.21)	0.163
Procedure-related adverse event	0.21 (0.05–0.90)	0.036
Reoperation	0.99 (0.51–1.92)	0.977

An odds ratio under 1.0 indicates a lower risk for the outcome in question for night-time versus daytime surgery and vice versa

Adjusted for study center, delay from diagnosis to surgery, age, sex, cardiac arrhythmia, preoperative Glasgow Coma Scale score, preoperative modified Rankin Scale, preoperative midline shift, preoperative hematoma width, preoperative use of antithrombotic medication

Analysis including 567 patients as the preoperative CT for one patient could not be retrieved

*OR*, odds ratio; *CI*, confidence interval

The sensitivity analysis using ordinal logistic regression showed an ordinal shift in mRS at six months favoring night-time surgery, but did not reach statistical significance (cOR 0.67, 95% CI 0.41–1.10, *p* = 0.112).

## Discussion

This posthoc analysis of a nationwide RCT indicated that night-time surgery for CSDH does not associate with poorer outcome than daytime surgery. Interestingly, it showed a significant association with fewer severe and procedure-related adverse events during night-time surgeries.

The findings of our study contribute to the ongoing discussion on the optimal timing of surgical interventions. Contrary to the traditional preference for daytime surgeries, our analysis suggests that night-time surgeries do not compromise patient outcomes in CSDHs and may even be associated with fewer adverse events. Several factors may contribute to this.

First, burr-hole drainage for CSDH is a relatively fast (median 26 min in the FINISH trial) [18] and simple procedure, and therefore may be less affected by the surgical team’s fatigue levels.

Second, in general, higher morbidity and mortality in night-time surgeries, have been associated with an increased need for blood transfusion and a higher rate of anesthesia handovers during these hours [2]. However, in CSDH, blood transfusions are very rarely needed. Additionally, most CSDH surgeries in Northern Europe are performed under local anesthesia, eliminating the need for anesthesia handovers altogether and reducing the possibility of anesthesia-related errors due to fatigue.

Third, performing the surgery as soon as reasonably possible, even during night-time, shortens the preoperative immobilization period and reduces decreased nutritional intake, both of which can be detrimental to elderly patients trying to recover from CSDH surgery.

Finally, surgeon experience may explain the difference in procedure-related adverse events. CSDH trepanation, often performed by trainees during the day, is a typical first neurosurgical procedure. Less experienced residents don't yet participate in night-time on-call duties, potentially biasing night-time operations towards more experienced surgeons and possibly contributing to fewer adverse events.

Recent meta-analyses have indicated increased morbidity and mortality associated with night-time surgeries across various specialties [4, 20]. These findings are further supported by a large cohort study involving 9,861 patients, which reported an increase in intraoperative adverse events during night-time surgeries [11]. In contrast, our study demonstrates a significantly lower rate of severe and procedure-related adverse events for night-time CSDH surgeries, suggesting that CSDH management presents a distinct risk profile from other surgical areas. This variance is likely attributed to the relative simplicity of CSDH drainage, typically performed under local anesthesia, unlike more complex abdominal surgeries, which have shown increased risks at night, such as anastomotic leaks in colorectal cancer surgeries and conversions to open surgery in laparoscopic cholecystectomies [3, 14].

An interesting finding in our study is the lower incidence of adverse events in the night-time group compared to the daytime group. One potential explanation could be a variance in patient demographics between the two groups. Patients undergoing surgery during the day may have broader comorbidities, not solely related to CSDH, thereby predisposing them to a greater risk of severe adverse events post-operatively. Operational scheduling may also play a role in this phenomenon. Daytime patients often experience extended preoperative hospitalizations, increasing their susceptibility to perioperative complications, including ischemic events, thrombosis, and infections [8]. In addition, many of the complications were quite rare, so the difference in group sizes would make it more likely for complications to be detected in the daytime group.

Similar to elderly patients with hip fractures, CSDH can be considered a sentinel health event, with associated high mortality [6, 13, 19, 22]. The main goal of CSDH evacuation surgery is to relieve neurological symptoms and improve quality of life, making timely intervention crucial. However, in some neurosurgical departments the organizational structures are such that daytime burr-hole surgery for CSDH is restricted due to competing elective surgery schedules. This can result in "emergent" CSDH cases being postponed until after elective surgeries or even until the next day if night-time procedures are avoided. Such delays, leading to prolonged immobilization and decreased nutritional intake, can be detrimental, adversely affecting the efficacy of CSDH surgery and reducing the likelihood of recovery [7, 16].

Our results suggest that burr-hole drainage for CSDH is effective and safe and should be done as soon as reasonably possible when clinically indicated. While our findings indicate potential advantages for night-time CSDH surgery, these must be interpreted within the context of broader operational practices and the potential for surgeon fatigue during more complex procedures. Thus, night-time CSDH surgeries should not be consistently avoided but rather integrated pragmatically into surgical planning.

## Limitations

This study provides valuable insights into the timing of CSDH surgery but is subject to several limitations. Notably, the proportion of night-time surgeries in our dataset was smaller than daytime surgeries, night-time surgeries accounting for only 17% of all procedures. This limited representation may restrict the generalizability of our findings and could introduce confounding by indication. The decision for night-time surgery reflects complex clinical judgments difficult to quantify, such as urgency based on subtle imaging findings (e.g., mass effect relative to brain atrophy) or direct link between CSDH and patient deterioration. These factors, alongside logistical considerations, may systematically differ between night-time and daytime surgeries, potentially biasing our results in ways challenging to account for statistically. We also lacked the data on the training status of the surgeons performing the operations. Differences in surgeon experience between daytime and night-time surgeries could influence outcomes, particularly the rate of procedure-related adverse events. Furthermore, our results may not be directly applicable to healthcare systems where these procedures are performed mainly under general anesthesia. Additionally, it is important to bear in mind that the post-hoc nature of this analysis means it was not initially designed to assess the impact of surgical timing on outcomes, potentially affecting the strength of the results.

## Conclusions

Our results suggest that the simple procedure of CSDH evacuation through a single burr-hole under local anesthesia can be as safely performed during night-time as during daytime. Night-time CSDH surgery was not associated with worse postoperative outcomes compared to daytime surgery. Our findings underscore the potential for flexible surgical scheduling to optimize patient outcomes. Night-time CSDH surgeries should not be viewed as a last resort but as a viable option within a broader strategy for managing surgical caseloads and enhancing patient care.

## Supplementary Information

Below is the link to the electronic supplementary material.

Supplementary file1 (PDF 152 KB)

## Data Availability

All data requests should be submitted to RR by email for consideration. Access to anonymised data may be granted following review and consideration of the Act on the Secondary Use of Health and Social Data in Finland.

## Declarations

We received written informed consent from the patients or their next-of-kin. The FINISH trial was approved by the ethics committee of Helsinki University Hospital (HUS/3035/2019 §238).
