# Correction to: Night‑time versus daytime surgical outcomes in chronic subdural hematomas: a post hoc analysis of the FINISH randomized trial

**DOI:** 10.1007/s00701-024-06352-z

**Published:** 2024-11-12

**Authors:** Elias Oulasvirta, Oula Knuutinen, Pihla Tommiska, Riku Kivisaari, Rahul Raj, on behalf of the FINISH study group, Abdirisak Ahmed, Tarmo Areda, Jiri Jr Bartek, Tomasz Czuba, Nils Danner, Antti-Pekka Elomaa, Janek Frantzén, Ilkka Haapala, Joonas Haapasalo, Juuso Heikkilä, Minttu Hellman, Henna Henttonen, Nora Huuska, Teppo L. N. Järvinen, Henna-Kaisa Jyrkkänen, Aku Kaipainen, Olli-Pekka Kämäräinen, Hanna Kämppi, Milla Kelahaara, Riku Kivisaari, Nikolai Klimko, Oula A. Knuutinen, Timo Koivisto, Tommi Korhonen, Janne Koskimäki, Anselmi Kovalainen, Xenia Kuparinen, Dan Laukka, Martin Lehecka, Kai Lehtimäki, Ville Leinonen, Kimmo Lönnrot, Antti Luikku, Teemu Luostarinen, Teemu Luoto, Janne Luotonen, Lauriina Lustig-Tammi, Henna-Riikka Maanpää, Jenni Määttä, Timo Möttönen, Eliisa Netti, Laura Nevaharju-Sarantis, Mika Niemelä, Tero Niskakangas, Mette Nissinen, Ville Nurminen, Minna Oinas, Teemu Ollonen, Anna Östberg, Elias Oulasvirta, Krista Pantzar, Katri Piilonen, Anni Pohjola, Markus Polvivaara, Jussi P. Posti, Rahul Raj, Linnea Rajala, Jonas Ranstam, Minna Rauhala, Behnam Rezai Jahromi, Miika Roiha, Ilkka Saarenpää, Antti Sajanti, Henrikki Salmi, Jarno Satopää, Christoph Schwartz, Niina Shemeikka, Pia Sorto, Simo Taimela, Sami Tetri, Tuomo Thesleff, Pihla Tommiska, Maarit Tuomisto, Nuutti Vartiainen, Ville Vasankari, Jyri Virta, Mikko Visuri, Paula Walle, Frederick A. Zeiler

**Affiliations:** 1 Department of Neurosurgery, Helsinki University Hospital and University of Helsinki, Bridge Hospital, HUS, Haartmaninkatu 4, Po. Box 340, 00029 Helsinki, Finland; 2 Department of Neurosurgery, Oulu University Hospital, Oulu, Finland

**Correction to: Acta Neurochirurgica (2024)**

10.1007/s00701-024-06302-9

The names of the FINISH study group are missing in the original manuscript.

**Complete list of FINISH investigators**

Ahmed, Abdirisak

 Areda, Tarmo 

 Bartek, Jiri Jr 

 Czuba, Tomasz 

 Danner, Nils

 Elomaa, Antti-Pekka 

 Frantzén, Janek 

 Haapala, Ilkka

Haapasalo, Joonas

 Heikkilä, Juuso 

 Hellman, Minttu 

 Henttonen, Henna 

 Huuska, Nora 

 Järvinen, Teppo LN 

Jyrkkänen, Henna-Kaisa 

 Kaipainen, Aku 

 Kämäräinen, Olli-Pekka 

 Kämppi, Hanna

 Kelahaara, Milla

 Kivisaari, Riku 

 Klimko, Nikolai

 Knuutinen, Oula A

 Koivisto, Timo

 Korhonen, Tommi 

 Koskimäki, Janne

 Kovalainen, Anselmi

 Kuparinen, Xenia

 Laukka, Dan

 Lehecka, Martin

 Lehtimäki, Kai

Leinonen, Ville

 Lönnrot, Kimmo

 Luikku, Antti

 Luostarinen, Teemu

 Luoto, Teemu

 Luotonen, Janne

 Lustig-Tammi, Lauriina

 Maanpää, Henna-Riikka

 Määttä, Jenni

 Möttönen, Timo

 Netti, Eliisa

Nevaharju-Sarantis, Laura

Niemelä, Mika

 Niskakangas, Tero

 Nissinen, Mette

 Nurminen, Ville

 Oinas, Minna

 Ollonen, Teemu

 Östberg, Anna

 Oulasvirta, Elias

 Pantzar, Krista

 Piilonen, Katri

 Pohjola, Anni

 Polvivaara, Markus

 Posti, Jussi P 

Raj, Rahul 

 Rajala, Linnea

Ranstam, Jonas

 Rauhala, Minna 

 Rezai Jahromi, Behnam 

Roiha, Miika

 Saarenpää, Ilkka

 Sajanti, Antti

 Salmi, Henrikki

 Satopää, Jarno

 Schwartz, Christoph

 Shemeikka, Niina

 Sorto, Pia

 Taimela, Simo

 Tetri, Sami

 Thesleff, Tuomo

 Tommiska, Pihla

 Tuomisto, Maarit

 Vartiainen, Nuutti

 Vasankari, Ville

 Virta, Jyri

 Visuri, Mikko

 Walle, Paula

 Zeiler, Frederick A

The original article has been corrected.

