# Supplementary material for: Night-time versus daytime surgical outcomes in chronic subdural hematomas: a post hoc analysis of the FINISH randomized trial
Source: Acta Neurochir (Wien). 2024 Oct 22;166(1):421. doi: 10.1007/s00701-024-06302-9 (PMC11496360; doi:10.1007/s00701-024-06302-9)
Supplement: Supplementary file 1 — Supplementary file1 (PDF 152 KB) [file 701_2024_6302_MOESM1_ESM.pdf]

eTable 1. Adverse events within six months for patient having surgery during daytime and night-time

Characteristic	Daytime surgery (n=490)	Nigh-time surgery (n=99)
Severe adverse events, no. (%)	94 (19.1%)	12 (12.1%)
Cardiac arrhythmia	2 (0.4%)	2 (2.0%)
Epileptic seizure	12 (2.5%)	2 (2.0%)
Gastrointestinal disorder*	6 (1.2%)	1 (1.0%)
Infection (systemic, requiring iv antibiotics)	43 (8.8%)	5 (5.1%)
Intracranial hemorrhage†	19 (3.9%)	1 (1.0%)
Intracranial ischemia	11 (2.3%)	1 (1.0%)
Myocardial infarction	1 (0.2%)	0 (0%)
New trauma (injury requiring hospitalization)	12 (2.5%)	0 (0%)
Other medical event requiring hospitalization ‡	6 (1.2%)	0 (0%)
Pulmonary embolism (or DVT causing PE)	8 (1.6%)	0 (0%)
Tension pneumocephalus	3 (0.6%)	1 (1.0%)
Minor adverse events, no. (%)	83 (16.9%)	14 (14.1%)
Cardiac arrhythmia	0 (0%)	1 (1.0%)
Infection (non-systemic, not prolonging hospitalization)	63 (13.0%)	12 (12.1%)
Local other wound problem	2 (0.4%)	0 (0%)
New trauma (not prolonging hospitalization)	14 (2.9%)	0 (0%)
Normal pressure hydrocephalus	4 (0.8%)	1 (1.0%)
Other medical event not requiring hospitalization or surgery	11 (2.3%)	1 (1.0%)
Skin disorder or rash	4 (0.8%)	0 (0%)
Thrombophlebitis, DVT not causing pe or chronic PE	5 (1.0%)	1 (1.0%)
Transient postoperative hemiparesis	1 (0.2%)	0 (0%)
Procedure-related adverse events, no. (%)	38 (7.8%)	2 (2.0%)
Acute subdural hematoma	3 (0.6%)	0 (0%)
Cerebral infarction	8 (1.6%)	0 (0%)
Critical limb ischemia	1 (0.2%)	0 (0%)
Deep venous thrombosis requiring hospitalization	2 (0.4%)	0 (0%)
Epileptic seizure	11 (2.3%)	1 (1.0%)
Intracerebral hemorrhage	1 (0.2%)	0 (0%)
Intracranial empyema	1 (0.2%)	0 (0%)
Mesenteric ischemia	2 (0.4%)	0 (0%)
Pulmonary embolism	1 (0.2%)	0 (0%)
Subarachnoid hemorrhage	1 (0.2%)	0 (0%)
Tension pneumocephalus	3 (0.6%)	1 (1.0%)
Problem with drain removal	1 (0.2%)	0 (0%)
Wound infection	2 (0.4%)	0 (0%)

One patient can have zero or several adverse events.

*bowel perforation, mesenteric ischemia, cholecystitis
†acute subdural hematoma, spontaneous or traumatic intracerebral hemorrhage, traumatic subarachnoid hemorrhage, growth of contralateral chronic subdural hematoma, new contralateral chronic subdural hematoma
‡ cardiac insufficiency, critical limb ischemia, transient ischemic attack
Abbreviations: DVT=deep venous thrombosis, PE=pulmonary embolism
